# Incidence of Delirium in Critically Ill Cancer Patients

**DOI:** 10.1155/2018/4193275

**Published:** 2018-07-08

**Authors:** Luis A. Sánchez-Hurtado, Nancy Hernández-Sánchez, Mario Del Moral-Armengol, Humberto Guevara-García, Francisco J. García-Guillén, Ángel Herrera-Gómez, Silvio A. Ñamendys-Silva

**Affiliations:** ^1^Department of Critical Care Medicine, Instituto Nacional de Cancerología, Mexico City, Mexico; ^2^Department of Critical Care Medicine, Hospital Especialidades Centro Médico Nacional La Raza, Mexican Institute of Social Security, Mexico City, Mexico; ^3^Department of Critical Care Medicine, UMAE Hospital de Traumatologia “Dr. Victor de la Fuente Narvaez”, Mexican Institute of Social Security, Mexico City, Mexico; ^4^Department of Critical Care Medicine, Instituto Nacional de Ciencias Médicas y Nutrición Salvador Zubirán, Mexico City, Mexico; ^5^Department of Critical Care Medicine, Medica Sur Clinic & Foundation, Mexico City, Mexico

## Abstract

**Objective:**

The aim of this study was to estimate the incidence of delirium and its risk factors among critically ill cancer patients in an intensive care unit (ICU).

**Materials and Methods:**

This is a prospective cohort study. The Confusion Assessment Method for the Intensive Care Unit (CAM-ICU) was measured daily at morning to diagnose delirium by a physician. Delirium was diagnosed when the daily was positive during a patient's ICU stay. All patients were followed until they were discharged from the ICU. Using logistic regression, we estimated potential risk factors for developing delirium. The primary outcome was the development of ICU delirium.

**Results:**

There were 109 patients included in the study. Patients had a mean age of 48.6 ± 18.07 years, and the main reason for admission to the ICU was septic shock (40.4%). The incidence of delirium was 22.9%. The mortality among all subjects was 15.6%; the mortality rate in patients who developed delirium was 12%. The only variable that had an association with the development of delirium in the ICU was the days of use of mechanical ventilation (OR: 1.06; CI 95%: 0.99–1.13;*p*=0.07).

**Conclusion:**

Delirium is a frequent condition in critically ill cancer patients admitted to the ICU. The duration in days of mechanical ventilation is potential risk factors for developing delirium during an ICU stay. Delirium was not associated with a higher rate of mortality in this group of patients.

## 1. Introduction

Delirium is an acute brain dysfunction, characterized by an acute change or fluctuation in mental status, inattention, disorganized thinking, or an alteration in level of consciousness [1–4]. A delirium assessment is a necessary routine task within an intensive care unit (ICU) because the presence of delirium is associated with morbidity, mortality, prolonged ICU hospitalization, increased time on a ventilator, and overall greater healthcare costs [5–7]. Delirium has long-term consequences, such as prolonged cognitive impairment, impaired activities of daily living, and decreased quality of life in survivors of a critical illness [8, 9]. Multiple risk factors for delirium, classified as predisposing and precipitating risk factors, include age, presence of a previous illness, high severity of acute illness, and therapeutic options such as mechanical ventilation, sedation, emergency surgery, and metabolic disorders, among others [5, 10].

The prevalence of delirium in the ICU setting has been reported to range from 20 to 40%; when mechanical ventilation is used, this rate increases to 60–80%. This wide range can be explained by a number of characteristics including illness severity and the instrument used. In specialized ICUs, the prevalence may be higher [2, 5]. Older critically ill patients may have a rate of more than 80% [9].

In oncological patients, delirium is the most common neuropsychiatric complication and has the same consequences as in critically ill patients without cancer [11, 12]. The reported incidence rate varies widely, from 10 to 85%, depending on the stage of cancer [12, 13]. However, when oncological patients are critically ill, there is very minimal information about the frequency of delirium. The vast majority of information on this topic has come from hospitalized or palliative oncological patients [13]. In this context, the aim of the current study was to describe the incidence of delirium and identify risk factors for its development among critically ill cancer patients who are hospitalized in an ICU.

## 2. Materials and Methods

### 2.1. Patient Population and Characteristics

A prospective, observational cohort study was conducted at the ICU of Instituto Nacional de Cancerologia located in Mexico City. We included all consecutive patients admitted to the ICU during the period between December 2015 and May 2016. The medical surgical ICU has nine beds exclusively for oncology patients. This study was approved by the institutional review board, and the need for informed consent was waived. Demographic and clinical data were collected using a paper form. The following information was collected: age, gender, ICU admission diagnosis, medical or surgical disorder, type of tumor (leukemia, lymphoma, or solid tumor), oncological disease status (recent diagnosis, active disease, complete remission, and others), disease stage (localized, metastatic, and others), type of treatment offered (chemotherapy, radiotherapy, or surgical), performance status [14] and the Karnofsky performance status scale [15], comorbidities, use of mechanical ventilation, diagnosis of acute renal failure, use and type of sedation, analgesic, steroids, antibiotics, and transfusion requirements. We calculated the Sequential Organ Failure Assessment (SOFA) [16] and the Mexican Sequential Organ Failure Assessment (MEXSOFA) score for evaluation of organic failures [17]. We measured the presence and severity of pain with the Numerical Rating for Pain Scale (NRP) or Behavioral Pain Scale (BPS), depending on the use of mechanical ventilation [18]. We recorded length of time before admission to the ICU (days), length of stay in the ICU stays (days), and subsequent hospital stay.

### 2.2. Delirium Assessment

The primary outcome was the development of ICU delirium, defined as a positive assessment for delirium during a patient's ICU stay. A delirium was diagnosed when the Confusion Assessment Method for the Intensive Care Unit (CAM-ICU) [1]. All the evaluations were performed by the same intensivist physician previously trained to perform the CAM-ICU measurement [1]. The patients were screened at the time of admission and each day in the morning (10:00–11:00 AM). According to the original description of CAM-ICU, presence of delirium, it was defined by changes in the next aspects: acute change or fluctuating course of mental state, inattention, altered level of consciousness, and disorganized thinking. The CAM-ICU aspect of the presence of acute changes or fluctuating course of mental state in the past 24 hours, through oscillation of the consciousness level according to the Richmond Agitation-Sedation Scale (RASS), all patients with RASS ≥ 3 (movement or ocular opening to the verbal call but without visual contact) were considered eligible for the evaluation of the presence of delirium. This evaluation can be performed in sedated patients and patients with no sedation, as well as in those submitted to mechanical ventilation. We do not perform the CAM-ICU if a patient is unconscious, comatose, or under deep sedation (i.e., RASS −4 or −5).

### 2.3. Statistical Analysis

Statistical analyses were performed using the Statistical Package for Social Sciences program (SPSS version 20.0, Chicago, IL). The Kolmogorov–Smirnov test was used to evaluate the distribution of variables. Continuous variables are reported as the mean ± SD or median (25–75% interquartile range (IQR)). Nominal variables are presented as percentages. Differences between groups were assessed using the chi-square test, Fisher's exact test, Student's *t*-test, or Mann–Whitney *U* test, as appropriate. Univariate and multivariate logistic regression analyses were used to identify factors associated with delirium. Variables with *p* values ≤ 0.200 in the univariate analysis or those considered clinically relevant (age, SOFA score, type of neoplasm and extension, comorbidities, and acute kidney injury) were entered into the multivariate analysis. In the multivariate model, *p* < 0.050 was used to denote statistically significant association between each variable and delirium. Results are presented as odds ratios (ORs) and their associated 95% confidence intervals (CIs). The area under the receiver operating characteristic curve was used to assess the discrimination of the model. Goodness of fit (Hosmer–Lemeshow) was calculated to assess the relevance of the logistic regression model (calibration), where *p* values > 0.05 indicate good model fit.

## 3. Results

During the period of study data collection, 109 patients were hospitalized in the ICU, and all were included in the analysis. The mean age was 49.25 ± 18.23 years old, and 62 (56.9%) were women. A total of 52 (47%) patients presented one or more comorbidities, and arterial hypertension and diabetes mellitus occurred most frequently. The main reason for ICU admission was septic shock (*n*=44, 40.4%), hypovolemic shock (*n*=25, 22.9%), and acute respiratory failure (*n*=12, 11%). In terms of the type of solid tumor, the principal organs affected were the colon (*n*=15, 13.8%), cervical uterus (*n*=13, 11.9%), esophagus (*n*=7, 6.4%), and ovaries (*n*=7, 6.4%). The demographic characteristics of the patients based on the presence of delirium are present in Table 1.

The incidence of delirium was 22.9% (*n*=25), and patients spent a median of 3 days (IQR: 1.5–7.5) in the ICU before the delirium onset. The most frequently observed subtype of delirium was mixed 44% (*n*=11), hypoactive delirium 24% (*n*=6), and hyperactive delirium 28% (*n*=7). The mortality among all subjects was 15.6% (*n*=17), and in patients who developed delirium, mortality was 12% (*n*=3).

After reviewing the potential risk factors and clinical characteristics under investigation in the current study, we found several significant differences between patients with delirium and those without delirium; more specifically, patients with delirium were found to be on mechanical ventilation for a greater number of day, were older in age, and their cancer had metastasized (Tables 1 and 2). These characteristics, as well as other characteristic deemed clinically relevant, were included in univariate and multivariate logistic regression models; results of the analyses indicate that mechanical ventilation for a greater number of day is associated with the development of delirium in the ICU (Table 3).

## 4. Discussion

Delirium is a frequent complication in patients hospitalized or admitted to the ICU. This study found that in patients admitted to a mixed oncology ICU, the incidence of delirium was 22.9%. The time in days of use of mechanical ventilation was the only characteristic associated with the development of delirium at the ICU when adjusted for another variable. Moreover, in the current sample of patients, there was no difference in mortality rate when delirium was present or absent.

Previously reported estimates of delirium incidence in critically ill patients ranged from 10 to 80%; this wide range depends on a factor such as the type of acute condition requiring ICU admission, with the highest incidence rates found in older patients and those who are in the postoperative period [6, 18, 19]. Prior researchers have theorized that the wide range of reported delirium incidence rates were a result of different methods for diagnosing delirium, as well as the clinical conditions. The results of our study, which was conducted in an oncological ICU, are similar to other non-oncological populations; moreover, this study also found lower rates of delirium than in studies with oncological patients on mechanical ventilation [5, 19].

There are a number of different risk factors for developing delirium in the ICU, including patients' age, severity of illness, use of sedatives, use of mechanical ventilation, preexisting conditions, and other factors [6]. In our study, several of these risk factors were frequently observed in patients with delirium, although there was not a significant statistical association; however, any of them showed a statistical association in patients with delirium.

The use of mechanical ventilation is a fully identified risk factor for the development of delirium, and the duration in days of this therapeutic strategy and support has also shown association [20, 21] as authors have shown in the same way as has been observed in our results, where it was the only variable with a weak association with development of delirium, despite the evident difference in the duration of the days of mechanical ventilation between subjects with delirium and those who did not develop it.

Oncological patients with hematological or solid neoplasm frequently present with many predisposing risk factors for delirium, including frailty, comorbidities, polypharmacy, and malnutrition; however, a limitation of the current study is that we did not measure these variables as potential confounders variables [12, 22].

Several studies have shown the consistent relationship between delirium and mortality in oncological and non-oncological patients, in both the ICU and a general hospital setting [5, 19]; however, in our study, we found a low overall rate of mortality, and there were no differences in the mortality rates of patients with and without delirium. Patients typically have a lower mortality rate when they spend less time on mechanical ventilation, spend less time in the ICU, and have a high proportion of solid tumor [23], which may explain the current findings. Our study found a lower patient mortality rate compared to other studies with critically ill cancer patients, despite similar patient organ failure in our study and previous studies, as measured by the SOFA score. Patients included in our study had better performance status, which may explain the difference in research finding [19].

This study has important limitations. First, the study was conducted in a single cancer center and has a small sample size. These limitations may affect the external validity of our results, limiting their utility in other ICU environments. Second, we did not consider the metastasis site (when it was present in patients), which may have been a potential confounding variable.

## 5. Conclusion

Delirium is a frequent condition in critically ill cancer patients admitted to the ICU. The duration in days of mechanical ventilation is a potential risk factor for developing delirium during an ICU stay. Delirium was not associated with a higher rate of mortality in this group of patients.

## Figures and Tables

**Table 1:** Demographic and clinical data of patients.

	With delirium	Without delirium	*p*
Number of patients, *n* (%)	25 (22.9)	84 (77.1)	
Age (years), median ± standard deviation (SD)	55.4 ± 17.55	47.42 ± 18.13	0.050^$^
Sex, *n* (%)			
Women	16 (64)	46 (54.8)	0.410^*∗*^
Comorbidities, *n* (%)	11 (44)	41 (48.8)	0.670^*∗*^
Arterial hypertension	4 (16)	14 (16.7)	0.930^+^
Diabetes mellitus	4 (16)	12 (14.3)	0.760^+^
Heart failure	6 (24)	22 (26.2)	0.830^*∗*^
Type of patient, *n* (%)			
Medical condition	12 (48)	34 (40.5)	0.500^+^
Surgical condition	13 (52)	50 (59.5)	
Place before admission to ICU, *n* (%)			
Emergency room	0	2 (2.4)	
Ward	15 (60)	42 (50)	
Operating room	10 (40)	40 (47.6)	
Reason for admission to ICU, *n* (%)			
Septic shock	11 (44)	33 (39.3)	0.670^*∗*^
Hypovolemic shock	2 (8)	23 (27.4)	0.060°
Acute respiratory failure	5 (20)	7 (8.3)	0.100^*∗*^
Postsurgical care	1 (4)	6 (7.14)	0.999°
Postcardiac arrest care	3 (12)	3 (3.57)	0.130^*∗*^
Type of malignancies, *n* (%)			
Solid tumor	14 (56)	61 (72.6)	0.110^*∗*^
Hematologic neoplasm	11 (44)	23 (27.4)	
Status of oncological disease, *n* (%)			
Recent diagnosis	10 (40)	38 (33.3)	0.540^*∗*^
Active disease	9 (36)	49 (58.3)	
Complete remission	6 (24)	7 (8.3)	
Extension of neoplasm, *n* (%)			
Local or regional	17 (15.6)	72 (66)	0.040^*∗*^
Metastatic	8 (7.3)	12 (11)	
Treatment for cancer, *n* (%)			
Surgical treatment	13 (52)	42 (50)	0.860^*∗*^
Chemotherapy	9 (36)	46 (54.8)	0.100^*∗*^
Radiotherapy	6 (24)	1 (1.2)	0.410^+^
Karnofsky score	80 (65–95)	80 (70–90)	0.780°
ECOG	1 (0.5–2.5)	1 (1–2)	0.770°
SOFA score at admission to ICU	7 (5–10.5)	7 (3.5–10)	0.450°
MEXSOFA score at admission to ICU	8 (6–11)	8 (4–11)	0.420°
Mortality in the ICU, *n* (%)	3 (12)	14 (16.7)	0.750^+^

ICU: intensive care unit; SOFA: Sequential Organ Failure Assessment score, MEXSOFA: Mexican Sequential Organ Failure Assessment score; IQR: interquartile range; ^$^Student's *t*; ^*∗*^chi-squared; ^+^Fisher's exact; °Mann–Whitney *U*.

**Table 2:** Risk factors for development of delirium.

	With delirium	Without delirium	*p*
Alcoholism, *n* (%)	3 (12)	15 (17.9)	0.760^+^
Smoking, *n* (%)	4 (16)	18 (21.4)	0.770^+^
Acute kidney injury at time of admission to the ICU, *n* (%)	16 (64)	37 (44)	0.080^*∗*^
Mechanical ventilation at admission to the ICU, *n* (%)	19 (76)	54 (64.3)	0.270^+^
Time with mechanical ventilation (days), median (IQR)	8 (4–11)	2 (1–10)	0.010^+^
Use of sedative in the ICU, *n* (%)	17 (68)	50 (59.5)	0.44^*∗*^
Use of benzodiazepines, *n* (%)	9 (36)	31 (36.9)	0.930°
Use of mixed sedation, *n* (%)	3 (12)	2 (2.4)	0.080^+^
Analgesic with opioids, *n* (%)	22 (88)	73 (87)	0.999^*∗*^
Numerical Rating for Pain Scale	2.5 (0.5–4.5)	2.5 (0–5)	0.860°
Behavioral Pain Scale	3 (3–3)	3 (3–3)	0.930°
Use of steroids in the ICU, *n* (%)	8 (32)	23 (27.4)	0.650^*∗*^
Use of vasopressor in the ICU, *n* (%)	18 (72)	57 (67.9)	0.690^*∗*^
Transfusion during stay in the ICU, *n* (%)	10 (40)	41 (48.8)	0.490^*∗*^
Use of antibiotic in the ICU, *n* (%)	25 (100)	78 (92.9)	0.330^*∗*^
Time before admission to the ICU (days), median (IQR)	4 (1–9.5)	2 (1–13.5)	0.510°
Time in the ICU (days), median (IQR)	7 (2-5-13)	2 (1–6.75)	0.007°

ICU: intensive care unit; IQR: interquartile range; ^*∗*^chi-squared; ^+^Fisher's exact; ° Mann–Whitney *U*.

**Table 3:** Risk factors for delirium in oncological critical ill patients, univariate and multivariate analysis.

	Univariate			Multivariate		
	OR	CI 95%	*p*	OR	CI 95%	*p*
*Time with mechanical ventilation (days)*	**1.06**	**0.99–1.13**	**0.070**	**1.06**	**0.99–1.13**	**0.070**
Time in the ICU (days)	1.07	1.01–1.14	0.020			
Hematologic neoplasm	2.08	0.82–5.25	0.040			
Metastasis	2.83	0.99–7.98	0.040			
Age (years)	1.03	0.99–1.05	0.050			
Hypovolemic shock	0.23	0.05–1.05	0.060			
Use mixed sedation	5.59	0.88–35.56	0.070			
Acute kidney injury at admission to the ICU	2.26	0.89–5.68	0.080			
Chemotherapy	0.46	0.18–1.17	0.100			
Acute respiratory failure	2.75	0.79–9.58	0.110			
Postcardiac arrest care	3.68	0.69–19.52	0.130			
Use of mechanical ventilation	1.76	0.63–4.88	0.270			
MEXSOFA score at admission to the ICU	1.05	0.93–1.18	0.410			
SOFA score at admission to the ICU	1.04	0.93–1.18	0.440			
Use of sedative in the ICU	1.44	0.56–3.72	0.440			
Postsurgical care	0.54	0.06–4.72	0.580			
Septic shock	1.21	0.49–2.99	0.670			
Analgesics with opioids	0.91	0.23–3.53	0.880			
Use of benzodiazepines	0.96	0.38–2.43	0.930			

ICU: intensive care unit; SOFA: Sequential Organ Failure Assessment score; MEXSOFA: Mexican Sequential Organ Failure Assessment score; CI: confidence interval; Hosmer–Lemeshow *χ*^2^  = 9.24, *p*=0.240; auROC: 0.69 (CI 95% 0.57–0.82), *p*=0.0100.
